# Genetic Identification of Edible Bird’s Nest in Thailand Based on ARMS-PCR

**DOI:** 10.3389/fgene.2021.632232

**Published:** 2021-03-08

**Authors:** Dongyong Lv, Yaohua Fan, Wanhua Zhong, Piyanuch Lonan, Kunfeng Liu, Maoyong Wu, Yina Wu, Yueliang Liang, Xiaoping Lai, Geng Li, Liangwen Yu

**Affiliations:** ^1^School of Nursing, Guangzhou University of Chinese Medicine, Guangzhou, China; ^2^Shenzhen Hospital of Integrated Traditional Chinese and Western Medicine, Guangzhou University of Chinese Medicine, Shenzhen, China; ^3^School of Physical Education and Health, Guangzhou University of Chinese Medicine, Guangzhou, China; ^4^Laboratory Animal Center, Guangzhou University of Chinese Medicine, Guangzhou, China; ^5^School of Pharmaceutical Sciences, Guangzhou University of Chinese Medicine, Guangzhou, China; ^6^Guangzhou Tongkang Pharmaceutical Co., Ltd., Guangzhou, China; ^7^Guangdong Provincial Key Laboratory of New Drug Development and Research of Chinese Medicine, Mathematical Engineering Academy of Chinese Medicine, Guangzhou University of Chinese Medicine, Guangzhou, China; ^8^Guangdong Yunfu Vocational College of Chinese Medicine, Yunfu, China

**Keywords:** edible bird’s nest, ARMS-PCR, ND2, genetic information, guanidinium isothiocyanate, *Aerodramus fuciphagus*, *Aerodramus maximus*

## Abstract

Edible bird’s nest (EBN) is a popular delicacy in the Asian Pacific region originating from Indonesia, Malaysia, Thailand and Vietnam, which consist of various potential medicine value in Traditional Chinese Medicine (TCM). Thailand is one of the main exporters of EBN. However, the genetic information of EBN, a key part of molecular biology, has yet to be reported in Thailand. It is necessary to explore the genetic information of EBN in Thailand based on a quick and simple method to help protect the rights and interests of consumers. This research aimed to systematically evaluate different methods of extracting EBN DNA to improve the efficiency of the analysis of cytochrome b (Cytb) and NADH dehydrogenase subunit 2 (ND2) gene sequences, the establishment of phylogenetic trees, and the genetic information of EBN in Thailand. Additionally, we aimed to develop a quick and simple method for identifying EBN from different species based on the genetic information and amplification-refractory mutation system PCR (ARMS-PCR). By comparing the four methods [cetyltrimethylammonium bromide (CTAB), sodium dodecyl sulfate (SDS), kit and guanidinium isothiocyanate methods] for EBN extraction, we found that the guanidinium isothiocyanate method was the optimal extraction method. Phylogenetic trees generated on the basis of Cytb and ND2 gene analyses showed that 26 samples of house EBN and 4 samples of cave EBN came from *Aerodramus fuciphagus* and *Aerodramus maximus*, respectively. In addition, to distinguish different samples from different species of *Apodiformes*, we designed 4 polymerase chain reaction (PCR) amplification primers based on the ND2 gene sequences of *A. fuciphagus* and *A. maximus*. The ARMS-PCR results showed band lengths for *A. fuciphagus* EBN of 533, 402, and 201 bp, while those for *A. maximus* EBN were 463, 317, and 201 bp. Collectively, the results showed that ARMS-PCR is a fast and simple method for the genetic identification of EBN based on designing specific original identification primers.

## Introduction

Edible bird’s nest (EBN), produced by *swiftlets* of the *Aerodramus* genus (mainly *Aerodramus fuciphagus* and *Aerodramus maximus*) is constructed from viscous, sticky secretions of mucin glycoprotein from a pair of sublingual glands beneath the tongue of *swiftlets* (Looi et al., 2017). EBN has been esteemed as a nutritious food since the Yuan dynasty in China, which consist of various potential medicine value in Traditional Chinese Medicine (TCM). It is widely distributed in Indonesia, Malaysia, Thailand, Huaiji, and other Southeast Asian regions along the Pacific. Generally, there are two classification of EBN: house nests (found in *swiftlet* houses) and cave nests (found in natural caves) (Liu et al., 2020). With the development of the house nest industry and newly discovered properties of EBN, including neuroprotection and pro-conceptive effects, the consumption of EBN is gradually increasing (Careena et al., 2018; Albishtue et al., 2019). However, the EBN market value is estimated to range from $1000 to $50 000 per kg according to the *swiftlet* species (Liu et al., 2020). To increase profits, some unethical suppliers introduce cheaper adulterants into EBN, including high-protein and high-carbohydrate substances (Huang et al., 2019).

With the increasing occurrence of adulteration, there has been widespread interest in developing simple, sensitive and accurate techniques to check for the quality and authenticity of EBN, including physical and chemical techniques. For example, thermal analysis methods, gel electrophoresis technology, Fourier transform infrared spectroscopy (FTIR), Raman spectroscopy and the analysis of EBN fiber microstructure using arrays are applicable with the goal of the rapid authentication of EBN (Yang et al., 2014; Shim et al., 2017; Guo et al., 2018; Dai et al., 2020). Separation techniques have also been widely used, such as gas chromatography and liquid chromatography, which can analyze the composition of amino acids, saccharides, peptides, the polyunsaturated fatty acids, saturated fatty acids and mono-unsaturated fatty acids (Chua et al., 2015; Wong et al., 2018; Lee et al., 2020). At present, molecular biology is the most precise method for checking the quality of EBN. The loop-mediated isothermal amplification (LAMP) assay and polymerase chain reaction-restriction fragment length polymorphism (PCR-RFLP) analysis could be applicable for authenticating EBN (Lee et al., 2019; Liu et al., 2020). However, these techniques of genetic identification require expensive instrumentation and tedious sample preparation. On the other hand, the analysis of genetic information is generally carried out on the basis of sample-specific genes (Lee et al., 2017). Although, previous research have showed that the EBN samples in Indonesia derived from *A. fuciphagus* while the EBN samples in Huaiji derived from *Apus nipalensis* (Lin et al., 2009). EBNs from different producing areas show difference in genetic information. Thailand is one of the main exporters of EBN. However, the genetic information of EBN in Thailand has yet to be reported. Thus, it is urgent to develop a reliable and systematic method to clarify genetic information on EBN in Thailand.

Recently, many research suggested amplification-refractory mutation system PCR (ARMS-PCR) is the most rapid, reliable and cost-efficient genotyping method to identify genetic information (Alyethodi et al., 2018; Chao et al., 2018; Jin et al., 2020). Compared with PCR-RFLP, ARMS-PCR can detect the single nucleotide polymorphisms (SNPs) by four primers using only one PCR and needn’t restriction enzymes, probes and expensive equipment (Jin et al., 2020). Therefore, we hypothesized that ARMS-PCR was a reliable and simple method to identify genetic information on EBN in Thailand.

## Materials and Methods

### Samples and Chemicals

A total of 33 samples were purchased from Thailand, Indonesia and China by Guangzhou Tongkang Pharmaceutical Co., Ltd. (Table 1). Morphological identification was performed by Professor Xiaoping Lai and Professor Geng Li (Table 2). All samples were deposited in the Laboratory Animal Center of Guangzhou University of Chinese Medicine for future use. Cetyltrimethylammonium bromide (CTAB), Tris-base, Triton X-100, dichloromethane, isopropanol and Polyvinylpyrrolidone (PVP) were from Sigma-Aldrich Co., Ltd. (St. Louis, MO, United States). Sodium dodecyl sulfate (SDS) and ethylenediamine tetraacetic acid (EDTA) were from Biosharp Co., Ltd. (Hefei, Anhui, China). Buccal swab DNA extraction kit was from TIANGEN BIOTECH Co., Ltd. (Beijing, China). Guanidine isothiocyanate was from Solarbio Co., Ltd. (Beijing, China). Tris-HCl buffer and Tris-saturated phenol were from Leagene Biotechnology Co., Ltd. (Beijing, China). Dithiothreitol (DDT) was from Beyotime Co., Ltd. (Shanghai, China). Isoamylol and ethanol were from Sinopharm Chemical Reagent Co., Ltd. (Ningbo, China).

**TABLE 1:** Edible brd’s nest sample information.

**Sample code**	**Habitat**	**Region**	**Area of Thailand**	**Nesting environment**
20190119THZ1 (Z1)	Thailand	Nakhon Pathom	Midland	House
20190119THZ2 (Z2)	Thailand	Samut Prakan	Midland	House
2019011THZ3 (Z3)	Thailand	Samut Sakhon	Midland	House
20190119THD1 (D1)	Thailand	Pattaya	East	House
20190119THD2 (D2)	Thailand	Rayong	East	House
20190119THD3 (D3)	Thailand	Chanthaburi	East	House
20190119THD4 (D4)	Thailand	Chon Buri	East	House
20190119THD5 (D5)	Thailand	Chachoengsao	East	House
20190119THD6 (D6)	Thailand	Trat	East	House
20190119THX1 (X1)	Thailand	Phetchaburi	West	House
20190119THX2 (X2)	Thailand	Prachuap Khiri Khan	West	House
20190119THX3 (X3)	Thailand	Ratchaburi	West	House
20190119THN2 (N2)	Thailand	Narathiwat	South	House
20190119THN3 (N3)	Thailand	Nakhon Si Thammarat	South	House
20190119THN4 (N4)	Thailand	Pattani	South	House
20190119THN5 (N5)	Thailand	Phatthalung	South	House
20190119THN6 (N6)	Thailand	Phuket	South	House
20190119THN7 (N7)	Thailand	Yala	South	House
20190119THN8 (N8)	Thailand	Satun	South	House
20190119THN9 (N9)	Thailand	Krabi	South	Cave
20190119THN10 (N10)	Thailand	Krabi	South	House
20190119THN11 (N11)	Thailand	Chumphon	South	Cave
20190119THN12 (N12)	Thailand	Nakhon Si Thammarat	South	House
20190119THN13 (N13)	Thailand	Phangnga	South	Cave
20190119THN14 (N14)	Thailand	Chumphon	South	House
20190119THN15 (N15)	Thailand	Songkhla	South	House
20190119THN17 (N17)	Thailand	Suratthani	South	House
20190119THN18 (N18)	Thailand	Ranong	South	House
20190119THN19 (N19)	Thailand	Phatthalung	South	Cave
20190119THN21 (N21)	Thailand	Phangnga	South	House
ID1	Indonesia	Indonesia	–	House
CNH2	China	Huaiji	–	Cave
CNH3	China	Huaiji	–	Cave

**TABLE 2:** The key points of edible bird’s nest morphological identification.

**Methods**		**EBN**	**Fake**
Outward appearance character	Optical identification	Appear in dense fiber and irregular thread texture.	Appear to be thick and “air-tight” texture.
	Stereoscopy identification	(1) Semitransparent and shiny. (6×)	(1) Opaque and matt. (6×)
		(2) Glassy-smooth surface. (6×)	(2) Flat surface thick fold-shaped pattern. (6×)
		(3) Numerous fine cracks. (50×)	(3) No fine cracks. (50×)
		(4) Small white bubbles scattered. (50×)	(4) Numerous small white bubbles. (50×)
Olfactory character		Natural “egg-like” or protein smell.	Fishy, sourly, acidic smell.
Tactile character		(1) Soak and pull.	(1) Low elasticity.
		(2) High elasticity.	(2) Easily tear and broken.
Soaking character		(1) Color will not fade.	(1) Color will fade.
		(2) Water remains clear.	(2) Water turns cloudy.

### DNA Extraction

All samples were ground in a mortar by freezing using liquid nitrogen. Then EBN powder was obtained and deposited at −80°C for further experiments. To develop an efficient and rapid DNA extraction protocol, four different methods for DNA extraction from EBN were evaluated. Total genomic DNA from the EBN samples was extracted using the CTAB method, SDS method, a buccal swab DNA extraction kit (kit method) and the guanidine isothiocyanate method (Attitalla, 2011; Kampmann et al., 2016; Green and Sambrook, 2018; Xia et al., 2019).

#### DNA Extraction via the CTAB Method

First, 25 mg of EBN powder from five samples such as N5, N6, ID1, CNH2, and CNH3, respectively, was dissolved at 65°C in 200 μL CTAB buffer (10 mM Tris-HCl, 0.8 mM EDTA, 0.49 M NaCl, 2% CTAB, 2% DTT, and 3% PVP) for 1 h. Then, an equal volume of phenol (Tris-saturated phenol, dichloromethane, isopropanol = 25:24:1, pH 8.0) was added to the supernatant. After centrifugation (10,000 r/min) for 10 min, the supernatant was collected, and an equal volume of dichloromethane/isoamylol (24:1) was added. Then, 5 M NaCl (1/20 of the volume) and absolute ethanol (2 times the supernatant volume) were added. The solution was sealed in an airtight manner and placed at −20°C for 30 min, followed by centrifugation at 7,000 r/min at 4°C for 5 min. The sediment was collected and washed with the following sequence: 70% 200 μL ethanol and 100% 200 μL ethanol. After air drying at room temperature, 30 μL of TE buffer (10 mM Tris-HCl and 1 mM EDTA, pH 8.0) was added to the samples, followed by incubation for 5 min, and the samples were then stored at −20°C.

#### DNA Extraction via the SDS Method

25 mg EBN powder (N5, N6, ID1, CNH2, and CNH3) was dissolved in 500 μL SDS buffer (10 M NaOH, 0.1 M EDTA, 0.1 M Tris-HCl, 0.5 M NaCl, 10% SDS, 2% DTT, and 3% PVP) and 200 μL TE buffer at 55°C for 1 h. Then, an equal volume of Tris-saturated phenol was added to the supernatant, followed by centrifugation (12,000 r/min) for 10 min. The supernatant was collected, and an equal volume of dichloromethane/isoamylol (24:1), 0.1 volume of 3 M sodium acetate and 2.5 times the volume of 100% ethanol were added successively. Then, the solution was sealed in an airtight manner and placed at −20°C for 120 min, followed by centrifugation at 12,000 r/min for 5 min. The sediment was collected and washed with 1 mL 70% ethanol. Finally, 30 μL TE buffer was added to the samples, followed by incubation for 5 min and storage at −20°C.

#### DNA Extraction With Buccal Swab DNA Extraction Kit

25 mg EBN powder (N5, N6, ID1, CNH2, and CNH3) was dissolved in 400 μL GA buffer and 20 μL protease K at 56°C for 1 h. Then, 400 μL GB buffer was added to the samples, which were placed at 70°C for 10 min. When the temperature of the solution dropped to room temperature, 200 μL of 100% ethanol was added. The solution and flocculent precipitate were removed from the adsorption column and centrifuged (12,000 r/min) for 30 s. Then, the sediment was washed in the following sequence: 500 μL GD buffer, followed by 600 μL PW buffer. After centrifugation at 12,000 r/min for 3 s, 30 μL TE buffer was added to the sediment, followed by incubation for 5 min and storage at −20°C.

#### DNA Extraction via the Guanidine Isothiocyanate Method

25 mg EBN powder (D1–6, Z1–3, X1–3, N2–15, N17–19, N21, ID1, CNH2, and CNH3) was dissolved in 200 μL TE buffer and 400 μL guanidine isothiocyanate buffer (5 M guanidine isothiocyanate, 0.1 M EDTA, 0.1 M Tris-HCl, 1.3% Triton X-100, and 3% PVP) at 55°C for 1 h. Then, 300 μL of saturated phenol and 300 μL of dichloromethane/isoamylol (24:1) were added to the solution. After centrifugation (13,000 r/min) for 10 min, the supernatant was collected, and an equal volume of dichloromethane was added. After centrifugation (13,000 r/min) for 10 min, isoamylol (0.8 volume) was added to the supernatant, which was then centrifuged (12,000 r/min) for 10 min. The sediment was collected and washed with 1 mL of 70% ethanol. Finally, the samples were dissolved in 25 μL TE buffer and stored at −20°C.

### Analysis of DNA Purity and Concentration

The purity and concentration of DNA play a vital role in PCR, which is an important method of molecular analysis. Therefore, we sought to screen the best method of DNA extraction among common methods including the CTAB method, SDS method, kit method and guanidine isothiocyanate method according to the DNA mass concentration and *A*_260__/__280_ ratio. The DNA concentration and the ratio of the absorbance at 260 and 280 nm (*A*_260__/__280_ ratio) were evaluated on the same day as DNA extraction using a NanoDrop Lite spectrophotometer (Thermo Fisher Scientific, Waltham, MA, United States) (Sales et al., 2020). The *A*_260__/__280_ ratio was used to evaluate the purity of the DNA.

### Polymerase Chain Reaction (PCR) Amplification and Sequencing

The inhibition of PCR amplification was detected for the EBN DNA obtained by the four different extraction methods. The cytochrome b (Cytb) gene and NADH dehydrogenase subunit 2 (ND2) gene were amplified by PCR with primers designed by Primer Premier 5.0 (Table 3). The amplification reaction system had a volume of 25 μL, including 1 μL of each primer (10 μM), 2 μL (100 ng) of genomic DNA, and 12.5 μL of 2× Master Mix (blue) buffer (Beyotime, Shanghai, China). The reaction mixture was placed into the PCR amplification instrument, and PCR amplification, was performed according to a previously reported procedure (Liu et al., 2020). The PCR conditions started with the initial denaturation at 94°C for 5 min, 40 cycles of 94°C for 30 s, 58°C for 90 s, and 72°C for 60 s, followed by a final extension at 72°C for 4 min. In addition, the size of the amplicons as shown in Table 3.

**TABLE 3:** Polymerase chain reaction primers.

**Name/species**	**DNA regions**	**Primers**	**Sequence (5′ → 3′)**	**Amplicons size**
EBN from artificial *swiftlet* houses	Cytb	Am-439bp-Foward	CCCACCCCCTCAAACATCTC	
		Am-439bp-Reverse	CCCACCCCCTCAAACATCTC	
	ND2	ND2-1bp-Foward	AAAATGATGGTTTAACCCCTTC	
		ND2-700bp-Reverse	GTTCAGGGTGAGGAATACGG	
		ND2-888bp-Reverse	GATTGAGATGACTGTGGC	
		ND2-681bp-Foward	ATTCCTCACCCTGAACACAAC	
		ND2-1076bp-Reverse	TAAGTAGAGGAGAGGATTATGGGGG	
EBN from cave	Cytb	Am-Cytb-494bp-Foward	AGCAACCACTGAGTAATAGCCTG	
		Am-Cytb-494bp-Reverse	AGATGGGAAATGGATGAGAAGG	
	ND2	Am-ND2-1bp-Foward	TGAACCCCTACGCCAAACTAA	
		Am-ND2-661bp-Reverse	GTGTTTAGGGTGAGGAATACGG	
		Am-ND2-460bp-Foward	TCACCACCATAGCTATTTCTTCTAC	
		Am-ND2-1034bp-Foward	GGAGGTGAGGATTATGGGGG	
ARMS-PCR to identify EBN from *Aerodramus fuciphagus*	ND2	Af/1-553bp-Foward	TCTAGCAATCATTGAATAATAGCCTGAGC	Common 533 bp
		Af/1-553bp-Reverse	AGAAGGTTAGTAGAGTCAGTTTGGGGTTG	
		Af/1-A-201bp-Foward	CACCATCCTCTTCATAACATCCACA	Common 201 bp
		Af/1-G-402bp-Reverse	GGTGAGGAGGGTTGGGTCTAGTTAC	Common 402 bp
ARMS-PCR to identify EBN from *Aerodramus maximus*	ND2	Am-ND2-463bp-Foward	CCCATTCCACTTCTGATTTCCAGAAGTCC	Common 463 bp
		Am-ND2-463bp-Reverse	TTTAGGTAGGAAGCCTGTTAGGGGTGGG	
		Am-ND2-A-317bp-Foward	TATTTCTTCTACCACCTTAGGGGGCGGA	Common 317 bp
		Am-ND2-G-201bp-Reverse	CGGACCTGTGTTTGGTTTAGTCCCCTC	Common 201 bp

The PCR products were detected by performing agarose gel electrophoresis in 0.5× TBE buffer for 25 min at 180 V. Then, the gels were placed on a gel imager to evaluate the effect of PCR amplification. The PCR solution was sent to Beijing Qingke Biotechnology Co., Ltd. for bi-directional DNA sequencing.

### Sequencing of Cytb and ND2 Gene Fragments, Sequence Comparison, and Phylogenetic Analysis

Low-mass sequences and primer regions were removed using GeneStudio v.2.2.0.0, and images of the peaks obtained by sequencing were spliced and proofread. Then, an online search in the NCBI database^1^ was performed to obtain the sequences that were the most similar to those of the samples. The Cytb sequences of *A. fuciphagus*, *A. maximus*, *Aerodramus fuciphagus germani* and *Amazilia tzacatl* were downloaded from NCBI and combined with the sample sequences to form dataset 1. In addition, the ND2 sequences of *A. fuciphagus*, *A. maximus*, *A. fuciphagus germani*, and *A. tzacatl* were downloaded from NCBI and combined with the sample sequences to form dataset 2. After alignment using MAFFT v7.308, gBlocks was used to identify conserved fragments in the datasets (Katoh and Standley, 2013). Then, to ensure the accuracy of the analysis, the displacement of each dataset was calculated with DAMBE v7.0 (Xia, 2018). In addition, the *X*-value of each base substitution model was calculated with jModelTest v2.17, and the optimum base substitution model was selected based on the Akaike information criterion (AIC) standard (Darriba et al., 2012). Finally, the evolutionary distance among the samples were determined with MEGA-X based on kimura 2-parameter model (K2P). Maximum likelihood (ML) trees were constructed using MEGA-X, the reliability of the topology was evaluated using bootstrap values derived from 1,000 repetitions. The ML trees were visualized on the ITOL server^2^.

### ARMS-PCR Analysis Based on the ND2 Gene

Traditional methods of genetic identification present several disadvantages, such as their time-consuming nature and the complicated steps involved. Thus, we designed primers according to the sequence of ND2 for quickly identifying EBNs produced by *A. fuciphagus* or *A. maximus*. Four pairs of primers were designed with Primer Premier 5.0 according to the ND2 sequence of *A. fuciphagus* downloaded at NCBI^3^. Then, the samples were amplified in an amplification reaction system of 20 μL, including 0.5 μL of each primer, 7 μL (350 ng) genomic DNA, 1 μL MgCl_2_, 2 μL dNTPs, 0.5 μL Taq DNA polymerase, and 7.5 μL of 2× Master Mix Buffer. The solution was placed in the PCR amplification instrument, and PCR amplification was performed. The PCR conditions started with the initial denaturation at 95°C for 5 min, 40 cycles of 95°C for 30 s, 65°C for 30 s, and 72°C for 60 s, followed by a final extension at 72°C for 10 min. Furthermore, the PCR products were evaluated by agarose gel electrophoresis in 0.5× TBE buffer for 50 min at 100 V. Then, the gels were placed in a gel imager to detect the results.

### Statistical Analysis

The results were analyzed with SPSS 22.0 software (SPSS, Inc., Chicago, IL, United States) and expressed as the mean ± standard deviation. The data were analyzed by one-way analysis of variance (ANOVA) and Least-Signification Difference (LSD). *P* < 0.05 was considered to indicate significant differences.

## Results

### Evaluation of the Four Methods of DNA Extraction

**FIGURE 1:**
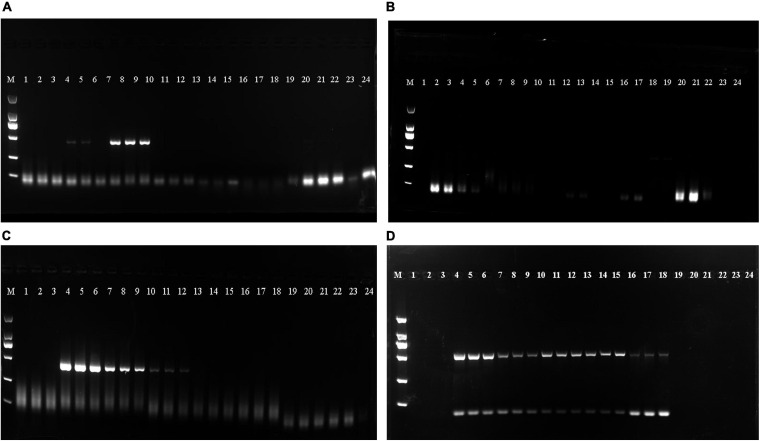
The Polymerase chain reaction (PCR) products obtained from the Cetyltrimethylammonium bromide (CTAB), Sodium dodecyl sulfate (SDS), buccal swab DNA extraction kit (Kit method) and guanidine isothiocyanate methods were checked on agarose gels. **(A)** PCR products from the CTAB method. **(B)** PCR products from SDS method. **(C)** PCR products from the kit method. **(D)** PCR products from the guanidine isothiocyanate method. Lanes 1–3: Porcine skin, lanes 4–6: N5, lanes 7–9: N6, lanes 10–12: ID1, lanes 13–15: CNH2, lanes 16–18: CNH3, lanes 19–21: agar, lanes 22–24: vermicelli.

As shown in Table 4, the DNA mass concentration and *A*_260__/__280_ ratio obtained following extraction via the guanidine isothiocyanate method were 51.381 ± 29.011 ng/μL and 1.654 ± 0.153, respectively. This revealed that the guanidine isothiocyanate method resulted in less protein and RNA contamination and was the most stable method for extracting EBN DNA. In addition, the results obtained from the four methods showed that all EBN DNA samples obtained via the guanidine isothiocyanate method were amplified successfully with a clear single band (Figure 1). These results suggest that the guanidine isothiocyanate method is suitable for extracting EBN DNA.

**TABLE 4:** Concentration and purity of extracted edible bird’s nest DNA determined with the cetyltrimethylammonium bromide, sodium dodecyl sulfate, buccal swab DNA extraction kit and guanidine isothiocyanate methods.

**Index**	**Cetyltrimethylammonium bromide (CTAB)**	**Sodium dodecyl sulfate (SDS)**	**Buccal swab DNA extraction kit (Kit method)**	**Guanidine isothiocyanate method**
*A*_260__/__280_	1.691 ± 0.488	1.163 ± 0.219^#^	1.086 ± 0.828^#^	1.654 ± 0.153
DNA mass concentration (ng/μ L)	155.888 ± 230.707^#^	143.846 ± 84.927^#^	48.521 ± 66.993	51.381 ± 29.011

**^#^P < 0.05 compared with the guanidine isothiocyanate method*.*

### Sequencing of Cytb and ND2 Gene Fragments

**FIGURE 2:**
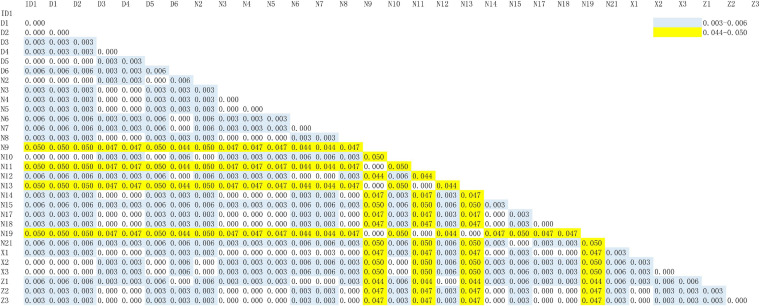
Kimura 2-parameter model distance of edible bird’s nest samples according to cytochrome b (Cytb) gene sequences.

**FIGURE 3:**
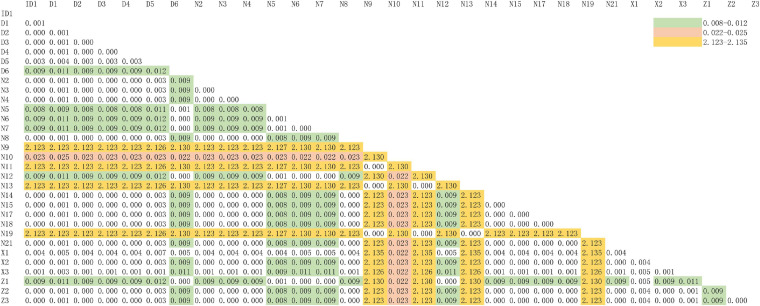
Kimura 2-parameter model distance of edible bird’s nest samples according to NADH dehydrogenase subunit 2 (ND2) gene sequences.

According to the higher copy number and faster evolutionary mutation rate of mitochondrial DNA (mtDNA) in EBN, the Cytb and ND2 genes are commonly used for genetic identification. Therefore, we explored a more suitable gene for ARMS-PCR analysis via Basic Local Alignment Search Tool (BLAST) and K2P evolutionary distance analysis. First, we evenly cut the sequences of the Cytb and ND2 gene fragments from all samples by using MEGA-X. Then, we obtained gene sequences of Cytb and ND2 with lengths of 356 and 726 bp, respectively. The BLAST results showed that the samples mainly came from two species, *A. fuciphagus* and *A. maximus*. According to the gene sequences of Cytb and ND2, samples Z1–Z3, D1–D6, X1–X3, N2–N8, N12, N14–15, N17–N21, and ID1 showed 97–100% similarity to *A. fuciphagus*, while samples N9, N11, N13, and N19 showed 97–100% similarity to *A. maximus* (Tables 5, 6). Subsequently, the results of K2P evolutionary distance analysis showed that the evolutionary distance between the Cytb sequences of the samples ranged from 0.003 to 0.050 (Figure 2). The distance between the ND2 sequences of the samples ranged from 0.001 to 2.135 (Figure 3). Samples Z1–Z3, D1–D6, X1–X3, N2–N8, N12, N14–15, N17–N21, and ID1 exhibited evolutionary distances ranging from 0 to 0.023, while samples N9, N11, N13, and N19 exhibited large evolutionary distances from other samples, which was consistent with the BLAST results.

**TABLE 5:** Basic local alignment search tool analysis results for cytochrome b (Cytb) gene sequences.

**Sample code**	**Most similar sequence GenBank accession no.**	**Max identity (%)**	**Expected value**	**Species**
D1	KX944187.1	100%	0	*Aerodramus fuciphagus*
D2	KX944187.1	100%	0	*Aerodramus fuciphagus*
D3	KX944196.1	100%	0	*Aerodramus fuciphagus*
D4	KX944196.1	99%	0	*Aerodramus fuciphagus*
D5	KX944187.1	100%	0	*Aerodramus fuciphagus*
D6	KX944196.1	100%	0	*Aerodramus fuciphagus*
ID1	KX944187.1	100%	0	*Aerodramus fuciphagus*
N2	KX944187.1	100%	0	*Aerodramus fuciphagus*
N3	KX944196.1	100%	0	*Aerodramus fuciphagus*
N4	KX944196.1	99%	0	*Aerodramus fuciphagus*
N5	KX944196.1	100%	0	*Aerodramus fuciphagus*
N6	KX944196.1	99%	0	*Aerodramus fuciphagus*
N7	KR818758.1	100%	0	*Aerodramus fuciphagus*
N8	KX944196.1	100%	0	*Aerodramus fuciphagus*
N10	KX944187.1	100%	0	*Aerodramus fuciphagus*
N12	KX944196.1	100%	0	*Aerodramus fuciphagus*
N14	KR818759.1	100%	0	*Aerodramus fuciphagus*
N15	KR818759.1	100%	0	*Aerodramus fuciphagus*
N17	KX944196.1	100%	0	*Aerodramus fuciphagus*
N18	KX944196.1	100%	0	*Aerodramus fuciphagus*
N21	KR818759.1	100%	0	*Aerodramus fuciphagus*
X1	KX944196.1	100%	0	*Aerodramus fuciphagus*
X2	KX944187.1	100%	0	*Aerodramus fuciphagus*
X3	KX944187.1	100%	0	*Aerodramus fuciphagus*
Z1	KR818758.1	100%	0	*Aerodramus fuciphagus*
Z2	KX944196.1	98%	0	*Aerodramus fuciphagus*
Z3	KX944196.1	100%	0	*Aerodramus fuciphagus*
N9	KR818764.1	100%	0	*Aerodramus maximus*
N11	KR818764.1	100%	0	*Aerodramus maximus*
N13	KR818764.1	100%	0	*Aerodramus maximus*
N19	KR818764.1	100%	0	*Aerodramus maximus*

**TABLE 6:** Basic local alignment search tool analysis results for NADH dehydrogenase subunit 2 (ND2) gene sequences.

**Sample code**	**Most similar sequence GenBank accession no.**	**Max identity (%)**	**Expected value**	**Species**
D1	KX944200.1	97%	0	*Aerodramus fuciphagus*
D2	KX944200.1	99%	0	*Aerodramus fuciphagus*
D3	KX944200.1	98%	0	*Aerodramus fuciphagus*
D4	KX944200.1	97%	0	*Aerodramus fuciphagus*
D5	KR905637.1	98%	0	*Aerodramus fuciphagus*
D6	KX944209.1	100%	0	*Aerodramus fuciphagus*
ID1	KX944200.1	98%	0	*Aerodramus fuciphagus*
N2	KX944200.1	98%	0	*Aerodramus fuciphagus*
N3	KX944200.1	99%	0	*Aerodramus fuciphagus*
N4	KX944200.1	100%	0	*Aerodramus fuciphagus*
N5	KR905637.1	99%	0	*Aerodramus fuciphagus*
N6	KR905637.1	100%	0	*Aerodramus fuciphagus*
N7	KR905637.1	100%	0	*Aerodramus fuciphagus*
N8	KX944209.1	100%	0	*Aerodramus fuciphagus*
N10	AY294491.1	99%	0	*Aerodramus fuciphagus*
N12	KR905637.1	99%	0	*Aerodramus fuciphagus*
N14	KX944209.1	99%	0	*Aerodramus fuciphagus*
N15	KX944209.1	100%	0	*Aerodramus fuciphagus*
N17	KX944200.1	98%	0	*Aerodramus fuciphagus*
N18	KX944200.1	98%	0	*Aerodramus fuciphagus*
N21	KX944209.1	100%	0	*Aerodramus fuciphagus*
X1	KX944200.1	100%	0	*Aerodramus fuciphagus*
X2	KX944200.1	99%	0	*Aerodramus fuciphagus*
X3	KX944200.1	98%	0	*Aerodramus fuciphagus*
Z1	KR905637.1	98%	0	*Aerodramus fuciphagus*
Z2	KX944200.1	100%	0	*Aerodramus fuciphagus*
Z3	KX944199.1	100%	0	*Aerodramus fuciphagus*
N9	AY294510.1	97%	0	*Aerodramus maximus*
N11	AY294510.1	97%	0	*Aerodramus maximus*
N13	AY294510.1	97%	0	*Aerodramus maximus*
N19	AY294510.1	97%	0	*Aerodramus maximus*

### Phylogenetic Analysis

**FIGURE 4:**
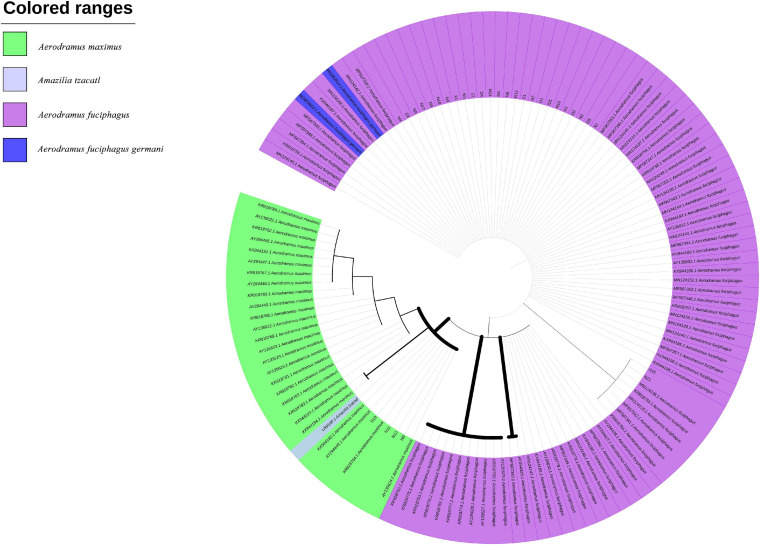
Phylogenetic tree of edible bird’s nest samples based on the maximum likelihood tree analysis of cytochrome b (Cytb) sequences.

**FIGURE 5:**
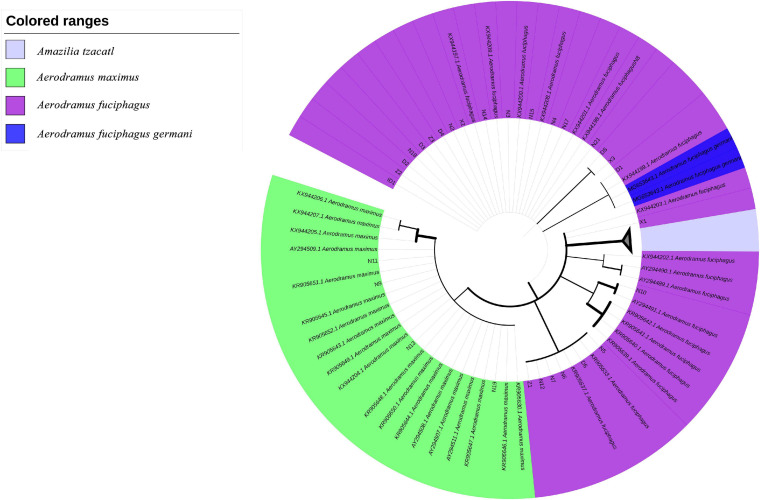
Phylogenetic tree of edible bird’s nest samples based on the maximum likelihood tree analysis of NADH dehydrogenase subunit 2 (ND2) sequences.

Furthermore, based on the Cytb and ND2 gene sequences, we constructed two ML trees to evaluate the evolutionary relationships among the EBNs. As shown in Figure 4, samples N9, N11, N13, and N19 were grouped with *A. maximus* at a confidence level of 98%, indicating that these samples almost certainly came from *A. maximus*. Samples N15 and N21 were grouped with *A. fuciphagus* and *A. fuciphagus germani* at a 53% confidence level. As shown in Figure 5, samples N9, N11, N13, and N19 were grouped with *A. maximus* at a confidence level of 66%. Samples Z1–Z3, D1–D6, X1–X3, N2–N8, N12, N14–15, N17–N21, and ID1 were grouped with *A. fuciphagus* and *A. fuciphagus germani* at a 71% confidence level, which indicates that these samples came from either *A. fuciphagus* or *A. fuciphagus germani*. The above results suggest that ND2, which contains more mutation sites than Cytb, is a more suitable gene for ARMS-PCR analysis to distinguish the genetic information of EBN.

### Genetic Identification of EBN Species via ARMS-PCR

**FIGURE 6:**
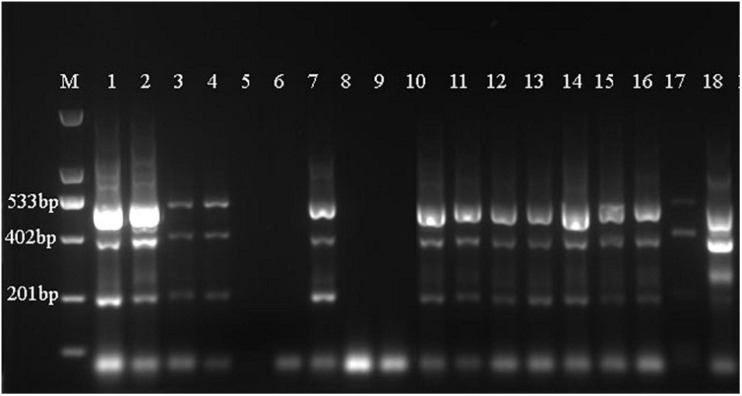
Amplification-refractory mutation system polymerase chain reaction (ARMS-PCR) profile. Analysis of genomic DNA from *Aerodramus fuciphagus* with the Af/1-553 bp-F/R primers. Lane M: 2,000 bp marker; lane 1 to lane 4: N5–N8; lane 5: N9; lane 6: N14; lane 7: N11; lane 8: N13; lane 9: N19; lanes 10–12: D1–D3; lanes 13–15: Z1–Z3; lanes 16–18: X1–X3. Samples N9, N11, N13, and N19 are from *A. maximus* in Thailand.

**FIGURE 7:**
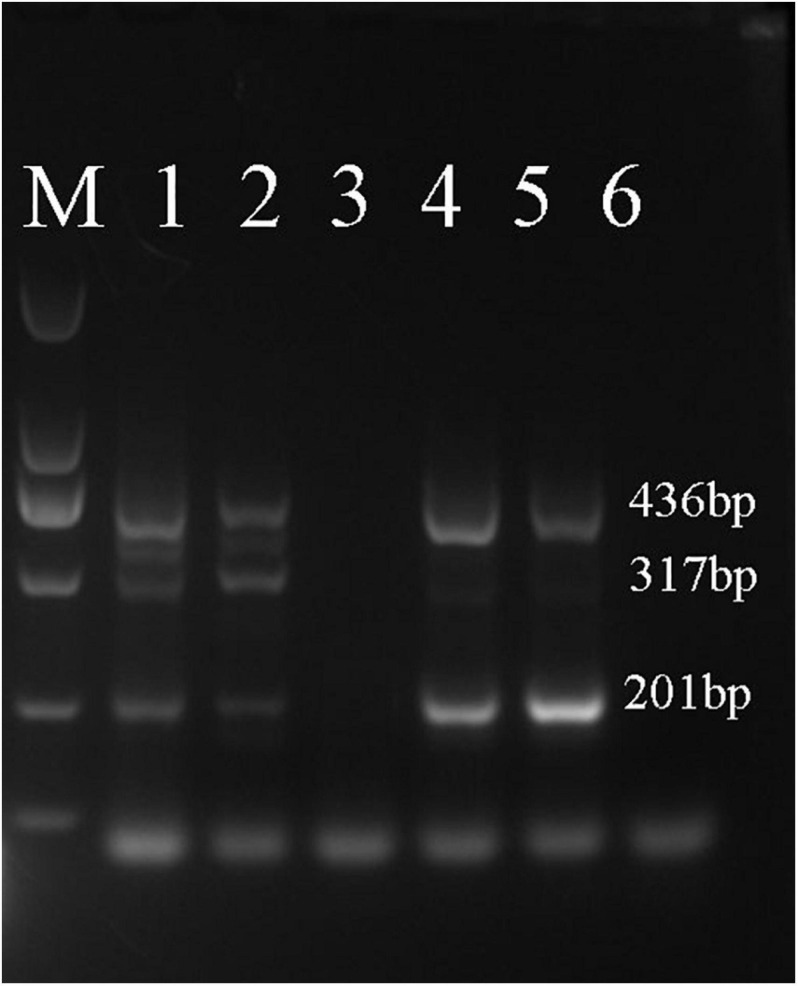
Amplification-refractory mutation system polymerase chain reaction (ARMS-PCR) profile. Analysis of genomic DNA from *A. maximus* with the Am-ND2-463 bp-F/R, Am-ND2-A-317 bp-F and Am-ND2-G-201 bp-R primers. Lane M: 2000 bp marker; lane 1: N9; lane 2: N11; lane 3: N5; lane 4: N13; lane 5: N19; lane 6: N6. Samples N5 and N6 are from *A. fuciphagus* in Thailand.

As shown in Figure 6, samples D1–3, Z1–3, X1–3, N5–8, and N14 were shown to belong to *A. fuciphagus*. The results suggested that the *A. fuciphagus* sequence could be digested into three fragments of 533, 402, and 201 bp, whereas the sequence of *A. maximus* did not follow the same pattern (Figure 6). In addition, compared with sample N5, samples N9, N11, N13, and N19 were identified to belong to *A. maximus* according to the visualization of three bands of 463, 317, and 201 bp (Figure 7).

## Discussion

Since EBN is considered a natural food product produced from *swiftlet*’s saliva which consist of various potential medicine value in Traditional Chinese Medicine (TCM). There is an urgent need to develop stable and efficient identification methods for EBN genetic identification. At present, sample-specific EBN genes that can be amplified and replicated by PCR are viable targets for the assessment of food quality. However, the efficient extraction and purification of DNA are vital for successful amplification (Vázquez-Novelle et al., 2005). Thus, we primarily assessed four different methods of DNA extraction. The guanidine isothiocyanate method is a rapid quantitative extraction method that does not rely on labor-intensive standard methodology (Lippke et al., 1987). Several reports have shown that the guanidine isothiocyanate method can extract proteins and RNA with significantly equivalent results (Monteiro et al., 2016; Siala et al., 2017; Joy et al., 2018). Our results also showed that the guanidine isothiocyanate method produced a clear single band in a shorter time than the other three methods (Figure 1). Therefore, we further distinguished the genetic information of EBN from Thailand by using the guanidine isothiocyanate method for DNA extraction.

Bioinformatics helps us understand complex biological problems by investigating similarities and differences that exist in polynucleic acids at the sequence level by using alignment algorithms, including dynamic programming and BLAST. BLAST is not only a sequence similarity search program but is also one of the most widely used bioinformatics research tools (Johnson et al., 2008). As shown by the BLAST, K2P distance and ML tree results, both the Cytb and ND2 genes can be used to genetically distinguish the source of EBNs (Figures 2–5 and Tables 5, 6). Furthermore, we identified 26 samples of house EBN in Thailand (Z1–Z3, D1–D6, X1–X3, N2–N8, N12, N14–15, N17–N21, and ID1) and 4 samples of cave EBN in Thailand (N9, N11, N13, and N19) belonging to *A. fuciphagus* and *A. maximus*, respectively. In accordance with the present results, previous studies have demonstrated that *swiftlets* producing white nest and living in *swiftlet* houses in Thailand should be considered members of a single panmictic population due to high gene flow between colonies and large population sizes (Aowphol et al., 2008). However, the above results based on the sequencing of Cytb and ND2 gene fragments could accurately genetically distinguish the EBNs from Thailand. This method is time consuming, has a high cost and cannot be generalized for application in the EBN market to identify EBN. On the other hand, the market value of different species differs greatly. The market values of *A. germani*, *A. fuciphagus*, *A. maximus*, and *Apus* EBNs are $46,000 per kg, $24,000 per kg, $15,000 per kg, and $10,000 per kg, respectively (Liu et al., 2020). Thus, there is an urgent need to develop simple, sensitive, accurate techniques to check the quality of EBNs.

ARMS-PCR is a simple, reliable and non-isotopic method that uses a set of 4 primers in a single PCR run to allow genotyping solely by the inspection of reaction mixtures after agarose gel electrophoresis (Newton et al., 1989; Rubio et al., 2008). The basis of ARMS-PCR is that each inner primer specifically matches one of the alleles associated with a SNP and includes a mismatch at position -2 from the 3′ terminus (Sauer et al., 2000). Recently, ARMS-PCR has been widely used to detect polymorphisms in genes such as NFKB1 ATTG, BRAF, and EGFR (T790M) (Chao et al., 2018; Li et al., 2019a, b). Thus, we further developed a novel method to distinguish the genetic information of EBN based on ARMS-PCR by using the ND2 gene, which exhibits more mutation sites than Cytb. First, we designed 2 primers located on either side of a SNP site and 2 primers with different upstream and downstream distances from the SNP site according to the ND2 gene sequences of *A. fuciphagus* and *A. maximus*. Then, we could rapidly identify EBNs derived from *A. fuciphagus* or *A. maximus* according to the size of the bands obtained from agarose gel electrophoresis. Consistent with the above results, samples D1–3, Z1–3, X1–3, N5–8, and N14 were found to belong to *A. fuciphagus*, for which three bands of 533, 402, and 201 bp were obtained upon agarose gel electrophoresis (Figure 6). Samples N9, N11, N13, and N19 were shown to belong to *A. maximus*, according to the visualization of three bands of 463, 317, and 201 bp (Figure 7). These results indicate that ARMS-PCR, a rapid, reliable and sensitive method for the genetic identification of EBNs between *A. maximus* and *A. fuciphagus*.

## Conclusion

The purpose of the current study was to develop a rapid, reliable and sensitive method for replenishing different genetic information of EBN in Thailand to identify EBN from different species. The first major finding was that the guanidine isothiocyanate method is suitable for extracting EBN DNA. The second major finding was that 26 samples of house EBN in Thailand and 4 samples of cave EBN in Thailand belonged to *A. fuciphagus* and *A. maximus*, respectively. Finally, we found that ARMS-PCR was a rapid, reliable, and sensitive method for the genetic identification of EBN.

## Data Availability Statement

The datasets generated for this study can be found in online repositories. The names of the repository/repositories and accession number(s) can be found in the article/Supplementary Material.

## Author Contributions

LY and DL designed the research. YF, PL, and KL performed the study. DL and WZ analyzed the data. YF and DL wrote the manuscript. GL and XL revised the manuscript. MW, YL, and YW provided technical support. All authors contributed to the article and approved the submitted version.

## Conflict of Interest

YL was employed by the company Guangzhou Tongkang Pharmaceutical Co., Ltd. The remaining authors declare that the research was conducted in the absence of any commercial or financial relationships that could be construed as a potential conflict of interest.

## Footnotes

1 https://blast.ncbi.nlm.nih.gov/Blast.cgi

2 https://itol.embl.de/

3 https://www.ncbi.nlm.nih.gov/nucleotide/
