# Supplementary material for: Genetic Identification of Edible Bird’s Nest in Thailand Based on ARMS-PCR
Source: Front Genet. 2021 Mar 8;12:632232. doi: 10.3389/fgene.2021.632232 (PMC7983251; doi:10.3389/fgene.2021.632232)
Supplement: Supplementary file 1 [file Table_1.DOCX]

Supplementary Material

# Supplementary Table 1

**Supplementary Table 1. The GenBank accession numbers for nucleotide sequences**

| **Sample code** | **GenBank accession number** | **Gene** | **Sample code** | **GenBank accession number** | **Gene** |
| --- | --- | --- | --- | --- | --- |
| 20190119THD2 | MW571009 | cytb | 20190119THD2 | MW570980 | ND2 |
| 20190119THD3 | MW571010 | cytb | 20190119THD3 | MW570981 | ND2 |
| 20190119THD4 | MW571011 | cytb | 20190119THD4 | MW570982 | ND2 |
| 20190119THD5 | MW571012 | cytb | 20190119THD5 | MW570983 | ND2 |
| 20190119THD6 | MW571013 | cytb | 20190119THD6 | MW570984 | ND2 |
| 20190119THN10 | MW571021 | cytb | 20190119THN10 | MW570992 | ND2 |
| 20190119THN12 | MW571022 | cytb | 20190119THN12 | MW570993 | ND2 |
| 20190119THN14 | MW571023 | cytb | 20190119THN14 | MW570994 | ND2 |
| 20190119THN15 | MW571024 | cytb | 20190119THN15 | MW570995 | ND2 |
| 20190119THN17 | MW571025 | cytb | 20190119THN17 | MW570996 | ND2 |
| 20190119THN18 | MW571026 | cytb | 20190119THN18 | MW570997 | ND2 |
| 20190119THN2 | MW571014 | cytb | 20190119THN2 | MW570985 | ND2 |
| 20190119THN21 | MW571027 | cytb | 20190119THN21 | MW570998 | ND2 |
| 20190119THN3 | MW571015 | cytb | 20190119THN3 | MW570986 | ND2 |
| 20190119THN4 | MW571016 | cytb | 20190119THN4 | MW570987 | ND2 |
| 20190119THN5 | MW571017 | cytb | 20190119THN5 | MW570988 | ND2 |
| 20190119THN6 | MW571018 | cytb | 20190119THN6 | MW570989 | ND2 |
| 20190119THN7 | MW571019 | cytb | 20190119THN7 | MW570990 | ND2 |
| 20190119THN8 | MW571020 | cytb | 20190119THN8 | MW570991 | ND2 |
| 20190119THX1 | MW571028 | cytb | 20190119THX1 | MW570999 | ND2 |
| 20190119THX2 | MW571029 | cytb | 20190119THX2 | MW571000 | ND2 |
| 20190119THX3 | MW571030 | cytb | 20190119THX3 | MW571001 | ND2 |
| 20190119THZ1 | MW571031 | cytb | 20190119THZ1 | MW571002 | ND2 |
| 20190119THZ2 | MW571032 | cytb | 20190119THZ2 | MW571003 | ND2 |
| 2019011THZ3 | MW571033 | cytb | 2019011THZ3 | MW571004 | ND2 |
| ID1 | MW571008 | cytb | ID1 | MW538959 | ND2 |
| 20190119THN9 | MW571034 | cytb | 20190119THN9 | MW570978 | ND2 |
| 20190119THN11 | MW571035 | cytb | 20190119THN11 | MW571005 | ND2 |
| 20190119THN13 | MW571036 | cytb | 20190119THN13 | MW571006 | ND2 |
| 20190119THN19 | MW571037 | cytb | 20190119THN19 | MW571007 | ND2 |
| 20190119THD1 | MW570979 | ND2 |  |  |  |
